# Genipin crosslinked chitosan/PEO nanofibrous scaffolds exhibiting an improved microenvironment for the regeneration of articular cartilage

**DOI:** 10.1177/08853282211002015

**Published:** 2021-03-17

**Authors:** Kuan Yong Ching, Orestis Andriotis, Bram Sengers, Martin Stolz

**Affiliations:** 1 Foundation, Study and Language Institute, University of Reading—Malaysia Campus, Iskandar Puteri, Malaysia; 2 Faculty of Engineering, National Centre for Advanced Tribology at Southampton, University of Southampton, Southampton, UK; 3 Institute of Lightweight Design and Structural Biomechanics, Vienna University of Technology, Vienna, Austria; 4 Bioengineering Science, Faculty of Engineering, University of Southampton, Southampton, UK

**Keywords:** Articular cartilage repair, tissue engineering, chitosan, poly(ethylene oxide), genipin, microenvironment, scaffold materials, mechanical environment

## Abstract

Towards optimizing the growth of extracellular matrix to produce repair cartilage for healing articular cartilage (AC) defects in joints, scaffold-based tissue engineering approaches have recently become a focus of clinical research. Scaffold-based approaches by electrospinning aim to support the differentiation of chondrocytes by providing an ultrastructure similar to the fibrillar meshwork in native cartilage. In a first step, we demonstrate how the blending of chitosan with poly(ethylene oxide) (PEO) allows concentrated chitosan solution to become electrospinnable. The chitosan-based scaffolds share the chemical structure and characteristics of glycosaminoglycans, which are important structural components of the cartilage extracellular matrix. Electrospinning produced nanofibrils of ∼100 nm thickness that are closely mimicking the size of collagen fibrils in human AC. The polymer scaffolds were stabilized in physiological conditions and their stiffness was tuned by introducing the biocompatible natural crosslinker genipin. We produced scaffolds that were crosslinked with 1.0% genipin to obtain values of stiffness that were in between the stiffness of the superficial zone human AC of 600 ± 150 kPa and deep zone AC of 1854 ± 483 kPa, whereas the stiffness of 1.5% genipin crosslinked scaffold was similar to the stiffness of deep zone AC. The scaffolds were degradable, which was indicated by changes in the fibril structure and a decrease in the scaffold stiffness after seven months. Histological and immunohistochemical analysis after three weeks of culture with human articular chondrocytes (HACs) showed a cell viability of over 90% on the scaffolds and new extracellular matrix deposited on the scaffolds.

## Introduction

Tissue engineering and regenerative medicine are concerned with the replacement or regeneration of cells, tissues, or organs to restore the normal biological function in the human body. Besides skin also articular cartilage was one of the first tissues of interest in tissue engineering because it only exhibits one single cell type (chondrocyte) and is avascular (lacks blood vessels), aneural (no neurons and nerves), and alymphatic (no lymphatic system). Due to its structural simplicity, AC was predicted to be one of the first tissues to be successfully regenerated but this was proven to be incorrect.^1^ A major hurdle in the engineering of AC is the de-differentiation of chondrocytes when exposed to a synthetic microenvironment, which often results in the development of calcified or fibrous cartilage exhibiting inferior functional properties and limits the production of functional cartilage.

The growth and remodeling of tissues are based on an ongoing, bidirectional interaction between cells and the extracellular matrix (ECM), in which the ECM exerts mechanical force directly on the cell membrane or indirectly putting force on the integrins. Both pathways are initiating cell-signaling cascades that produce changes in gene expressions, whereas cellular changes, in turn, affect the composition and structural arrangement of the ECM.^2,3^ To regrow functional AC, it is, therefore, important to provide the cells with their appropriate microenvironment, including the ultrastructure, stiffness and growth factors to control the cell fate and direct tissue development. Cells have been found to grow and maintain their function within a structure similar to their native extracellular matrix, but they lose their function when placed into a structure different from their native extracellular matrix.^4^ Hence, it is vital to provide the cells with a microenvironment that mimics the native AC and favours neo-cartilage growth.^5^

**Figure 1.**
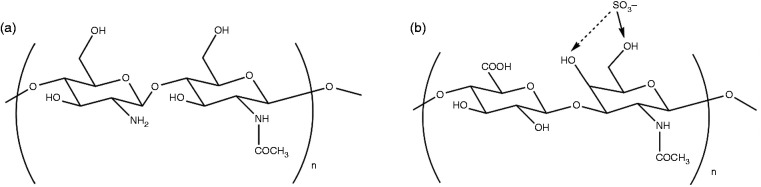
Structure of (a) chitosan and (b) chondroitin sulfate, the primary glycosaminoglycan present in AC.

Chitosan is the primary structural polymer in arthropod exoskeletons, shells of crustaceans, or the cuticles of insects. It is a polysaccharide made of amino sugars and is technically widely used because of its biocompatibility.^6–8^ Chitosan is composed of randomly distributed β-(1,4) linked D-glucosamine and N-acetyl-glucosamine (shown in Figure 1(a)). Interestingly, its chemical structure shares some characteristics with the chondroitin sulfate in AC (see Figure 1(b)), a type of sulfated glycosaminoglycans composed of repeating disaccharide structures of N-acetyl-galactosamine and glucuronic acids. Chondroitin sulfate is an important structural component of the cartilage ECM that modulates the chondrocytes morphology, differentiation, and function. Chitosan contains free amino groups that will be protonated under acidic conditions (pH < 6) to produce a positive charge, which allows for its ionic interactions with the negatively charged chondroitin sulfate,^9^ chondrocytes,^10,11^ growth factors, and cytokines,^12,13^ and hence, increases the biomimicry of chitosan *in vivo* environment. However, one of the main characteristics that limits the use of chitosan as scaffold materials for tissue engineering is its rigid and brittle nature, which makes it prone to rupture when loaded. Therefore, the mechanical integrity of chitosan needs to be improved for application as pre-engineered cartilage constructs.

The morphology and ductility of chitosan can be improved by blending or copolymerizing with other polymers, preferably uncharged polymers, which can prevent strong interactions between components.^14^ A blending approach is an easy and cost-effective method of combining at least two polymers to achieve specific properties, and the process allows rapid mixing of the system without large energy consumption and the potential to avoid unfavourable chemical reactions.^15^ The properties of the blends can be manipulated according to their end use. A well-known uncharged polymer that is widely used for blending with other biopolymers is poly(ethylene oxide) (PEO). It is one of the few synthetic polymers with the Food and Drug Administration (FDA) approval ^16^ and proven to be able to evoke cell proliferation and extracellular matrix generation, both *in vitro* and *in vivo.*^17–19^ It exhibits a structure of which each ethylene oxide unit is capable of binding with two to three water molecules.^20^ This creates a hydrophilic entrapment for the hydrophobic chitosan,^21^ rendering the chitosan/PEO blends highly hydrated, which is a feature needed for the transportation of nutrients in AC. Based on the intermolecular interactions between the various components that are occurring mainly due to hydrogen bonds and electrostatic interactions, chitosan/PEO blends with tailored chemical and mechanical properties can be created. Furthermore, blending PEO with chitosan will inevitably reduce the brittle nature of chitosan. This is because PEO with ultra-high molecular weight (>1 million Da) exists in flexible long chains that can serve to transfer load more effectively to the polymer backbone, thus, allowing its application as a structural support.^14,22^

Chitosan and PEO are both unstable and soluble in physiological buffers and, therefore, crosslinking agents are needed to bind the polymer network together to prevent the individual kinetic chains from dissolving into the surrounding solution. Chemical crosslinking agents react primarily with the amine groups on amino acids or proteins. Various chemical crosslinking agents, e.g., epoxy compounds, aldehydes, and carbodiimides, have been developed to stabilize the degradation of chitosan and tailor their mechanical properties, with glutaraldehyde probably being the most often used crosslinking agent in biomedical sciences.^23^ However, these chemical reagents are cytotoxic and may impair the biocompatibility of the crosslinked biomaterials. For example, it has been reported that even prolonged washing of 60 minutes was insufficient to remove the cytotoxic effects of glutaraldehyde due to its slow leaching behaviour.^24^ Therefore, much interest has been directed towards the naturally derived crosslinking agent, i.e., genipin, which is a crosslinker isolated from the *Gardenia jasminoides Ellis* fruit, and has been used in traditional Chinese medicine. Genipin was originally identified as a protein crosslinking agent, which reacts with free amino groups through oxygen radical-induced polymerization and dehydrogenation.^25,26^ It has been investigated as a crosslinking agent for biomaterials, e.g., chitosan,^27–29^ collagen,^29^ gelatin ^27^ and silk fibroin.^28^ The biocompatibility of genipin has been proven to be better than synthetic crosslinking agents such as glutaraldehyde, formaldehyde, and epoxy compounds.^30,31^ Indeed, genipin was about five to ten thousand times less cytotoxic and evoked 5000 times more cell proliferation than glutaraldehyde.^32,33^ It has also been shown that tissues and scaffolds crosslinked by genipin exhibit improved tensile strength and toughness, compared to glutaraldehyde and epoxy crosslinkers.^32–34^ Hence, genipin was used as the crosslinking agent to reduce the solubility of chitosan/PEO blends before they can serve as scaffold materials in physiological conditions, and to achieve the desired mechanical properties.

Since a microenvironment that mimics native cartilage is vital for the differentiation of chondrocytes, an ultrastructure comparable to the fibrillar meshwork in native cartilage can be obtained by electrospinning. The blending of chitosan with PEO has an added advantage, in this case as it allows the concentrated chitosan solution to become electrospinnable as a result of the enhanced chain entanglement due to the formation of hydrogen bonding between amino hydrogen (-NH_2_) from chitosan and oxygen (-C-O-C-) from PEO.^35,36^ The electrospun chitosan/PEO nanofibrous scaffolds, which mimic the collagen fibril meshwork of collagen, may serve as temporary cell matrices to transmit the tensile loads, whereas the glucosamine chemistry of chitosan could support the compressive stresses, mimicking the roles of fibrous collagen and glycosaminoglycan in the natural extracellular matrix.^37^ We hope to be able to tune the structural and mechanical properties of chitosan by combining it with the correct proportion of PEO and genipin crosslinker, thus, allowing it to serve as a structural support for the attachment of chondrocytes and lead to the generation of functional cartilage.

## Materials and methods

### Fabrication of chitosan/PEO nanofibrous scaffolds

Chitosan from crab shells with degree of deacetylation >85% and molecular weight of 190,000–310,000 Da (middle viscous, 28191; Sigma-Aldrich, Gillingham, UK) and PEO with molecular weight >5 MDa (A15536; Alfa Aesar, Lancaster, UK) were blended at a weight ratio of 1:0.33 and dissolved in aqueous solvent of 3.0% (w/w) acetic acid (33209; Sigma-Aldrich). Co-solvents containing 10% (w/w) of dimethyl sulfoxide (DMSO, D4540; Sigma-Aldrich) and 0.3% (w/w) of Triton X-100 (437002 A; VWR International, Poole, UK) were added to improve the electrospinning condition. The blend solutions were stirred at room temperature for 24 hours until viscous transparent solutions were obtained. Electrospinning was employed using a solution flow rate of 2 μl/min. Voltage supply and distance between the spinneret and collector were adjusted until a stable jet was obtained.

The electrospun chitosan/PEO scaffolds were then immersed in genipin solution for crosslinking reaction to occur. To prepare genipin solution, genipin powder (98%; Challenge Bioproducts, Yun-Lin Hsien, Taiwan) was dissolved in aqueous solution of 90% ethanol to obtain concentrations of 0.5%, 0.8%, 1.0% and 1.5% (w/w). A minimum concentration of 0.5% was used as a requirement to ensure complete crosslinking.^38^ The completion of crosslinking reaction was indicated by a colour change of the scaffolds from white to greenish blue, which occurred after a period of approximately 14 days. The crosslinked scaffolds were rinsed with excess of deionised water and immersed in water overnight to eradicate excess or unreacted genipin.

### Preparation of human articular cartilage

Human AC sample was obtained from the femoral head of haematologically normal osteoarthritic patient (age: 80 years) undergone total hip replacement surgery at the Southampton General Hospital, with the approval of the Southampton General Hospital and South West Hants Local research Ethnics Committee (LREC 194/99/1 and 210/01). Cartilage samples were taken from areas with no apparent signs of damage or disease and stored in minimum essential medium alpha (α-MEM, 11900–073; Life Technologies, Paisley, UK) on ice or at 4 °C until use.

### Scanning electron microscopy

The structure of scaffolds and human AC was investigated by scanning electron microscopy (SEM, JSM-6500F; JEOL, Tokyo, Japan) after enzymatic depletion of the proteoglycan moiety and chondrocytes in phosphate-buffered solutions (PBS, H15–002; PAA Laboratories, Pasching, Austria) containing 1 mg/ml bovine hyaluronidase (type I, H3506; Sigma-Aldrich) and 1 mg/ml trypsin (Trypsin-EDTA, T4174; Sigma-Aldrich) at 37 °C for three days. Specimens were then fixed with 4% paraformaldehyde (P/0840/53; Fisher Scientific, Loughborough, UK) in PBS for 4 hours at room temperature, rinsed with water and dehydrated in graded ethanol series.

Prior to SEM imaging, all chitosan/PEO scaffolds and cartilage samples were coated with a thin conducting layer of gold (∼10 nm) with a Hummer 6.2 Sputter System (Anatech, Union City, CA). Images were obtained using an emission current of 10 µA and an accelerating voltage of 10 kV.

### Indentation-type atomic force microscopy

A micrometre-sized spherical indenter made of borosilicate glass (radius, *r* = 5 µm, 02715-AB; SPI Supplies, West Chester, PA) was used to measure the overall stiffness properties of the various structural elements composing AC, as well as the overall stiffness contributed by the electrospun fibril meshwork. The spherical probe was glued onto tipless rectangular cantilever (spring constant, *k_c_* ∼6 N/m, type All In One-TL; BudgetSensors, Sofia, Bulgaria). Measurements by indentation-type atomic force microscopy (IT-AFM; MFP-3D; Asylum Research, Santa Barbara, CA) were carried out in PBS solution following protocols developed by our group.^39–41^ A maximum deflection of 100 nm was employed, corresponding to a load of ∼600 nN. Cyclic load-displacement curves were recorded at 0.5 Hz. Each individual data set consisted of 1024 load-displacement curves in a 32 × 32 curve grid, covering a sample area of 30 × 30 µm, at three different locations. The indentation depths were less than 10% of the overall sample thickness. The stiffness of sample, *E*, was calculated from equation (1).

$$\;E=\;\frac{\sqrt{\pi }}{2}\;\left(1-v^{2}\right)\;\frac{S}{\sqrt{A}}\;(1)$$

where *v* is the Poisson’s ratio of the sample, *S* is the contact stiffness with the dimension of force per unit depth, *A* is the projected contact area.

### Degradation of chitosan/PEO nanofibrous scaffolds

The degradation of scaffolds was tested in α-MEM medium at 37 °C. The medium was changed every 2 days. The structure and stiffness of the scaffolds was examined by SEM and IT-AFM, respectively, after one and seven months of degradation.

### Biocompatibility of chitosan/PEO nanofibrous scaffolds

The biocompatibility of scaffolds was tested with HACs, which were isolated from the cartilage specimens following digestion in trypsin-EDTA for 30 minutes, 1 mg/ml hyaluronidase for 15 minutes and 10 mg/ml collagenase B (11088831001; Roche Diagnostics, West Sussex, UK) for 15 hours. The isolated HACs were then expanded in α-MEM supplemented with 10% fetal bovine serum (FBS, 10270–106; Life Technologies), 100 unit/ml penicillin, 100 μg/ml streptomycin (Penicillin-Streptomycin, P4333; Sigma-Aldrich) and 100 μM L-ascorbic acid 2-phosphate sequimagnesium salt hydrate (A8960; Sigma-Aldrich). The HACs were harvested at confluence by trypsinization after one passage. Scaffolds were sterilised with absolute ethanol, rinsed with PBS, and seeded with HACs at a density of ∼7 × 10^5^ cells/cm^2^. The HACs-seeded scaffolds were cultured in chondrogenic induction medium made up of α-MEM supplemented with 10 ng/ml recombinant human transforming growth factor-β3 (TGF-β3, 100-36E; Peprotech, London, UK), 100 μM L-ascorbic acid 2-phosphate sequimagnesium salt hydrate, 10 nM dexamethasone (D4902; Sigma-Aldrich), and Insulin-Transferrin-Selenium-G (ITS-G, 41400; Life Technologies). All tissue cultures were incubated at 37 °C in humidified atmosphere with 5% CO_2_. Media were exchanged every two days. The constructs were harvested for live/dead cell staining, histological and immunohistochemical analysis after three weeks.

#### Live/dead cell staining

Metabolically active and necrotic cells were labelled with 10 µg/ml Cell Tracker Green CMFDA (5-chloromethylfluorescein diacetate, C7025; Life Technologies) and 5 µg/ml Ethidium Homodimer-1 (E1169; Life Technologies), respectively, for an hour. The constructs were incubated in tissue culture medium for 45 minutes and then fixed with 4% paraformaldehyde in PBS. The specimens were embedded in paraffin wax and sequential sections were cut in sections of 7 μm. After de-waxing with Histoclear (HS200; National Diagnostics, Leicestershire, UK) and re-hydration through graded ethanol, cell nuclei were stained with DAPI (4’,6-diamidino-2-phenylindole, dilactate, D3571; Life Technologies). Fluorescent images were acquired with Axiovert 200 microscope (Carl Zeiss Microscopy, Jena, Germany) equipped with Zeiss AxioCam HR colour camera, AxioCam MR3 monochrome camera and the Zeiss AxioVision 4.6 software.

#### Histology and immunohistochemistry

After de-waxing with Histoclear and re-hydrating through graded ethanol, cell nuclei were counter-stained with haematoxylin (H/0010/46; Fisher Scientific). The proteoglycan content was stained with 5 mg/ml Alcian blue 8GX (40046–0100; Acros Organics, Geel, Belgium) while total collagen content was stained with 10 mg/ml Direct Red 80 (365548; Sigma-Aldrich).

The expression of SOX-9, aggrecan, collagen I, II and X in the neo-cartilage were detected by immunohistochemistry. For anti-SOX-9 and anti-aggrecan antibodies, sections were treated with heat-induced antigen retrieval in 0.01 M citrate buffer at 60°C for 25 minutes, followed by quenching endogeneous peroxidase with 3% hydrogen peroxide (H_2_O_2_) and blocking with 1% bovine serum albumin (A3294, Sigma-Aldrich) in PBS. For anti-collagen I, II and X antibodies, sections were quenched with 3% H_2_O_2_, incubated with 520 μg/ml bovine hyaluronidase at 37 °C for 20 minutes and then blocked with 1% bovine serum albumin. Next, sections were incubated with the relevant primary antibody at 4 °C for 18 hours. The anti-SOX-9 (AB5535; Millipore, Billerica, MA) and anti-aggrecan (ab36861; Abcam, Cambridge, UK) antibodies were used at dilution of 1:150, anti-collagen I antibody (gift from Larry W. Fisher, National Institutes of Health, Maryland) at 1:1000, anti-collagen II (ab34712; Abcam) and anti-collagen X (234196; Millipore) antibodies at 1:100. This was followed by incubation with biotinylated secondary antibody (B7389; Sigma-Aldrich) at a dilution of 1:100 for an hour. Visualization of the immune complex involved the avidin-biotin method linked to peroxidase (ExtrAvidin Peroxidase, E2886; Sigma-Aldrich) at a dilution of 1:50 and 3-amino-9-ethylcarbazole (AEC, 132–32-1; Acros Organics, New Jersey). The reaction products were reddish brown in colour. Negative controls (omission of the primary antibody) were included in the control sections. Images were captured using an inverted light microscope (type BX51, Olympus Corporation, Tokyo, Japan) equipped with a dotSlide virtual slide system.

## Results

### Structure of chitosan/PEO nanofibrous scaffolds

**Figure 2.**
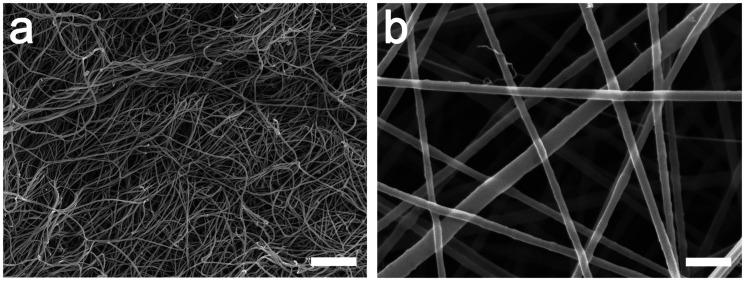
SEM micrographs comparing collagen fibril meshwork with electrospun fibril meshwork. (a) The collagen fibril meshwork of human AC after proteoglycan extraction consists of a pseudo-random meshwork of smooth collagen fibrils. (b) The fine and straight electrospun chitosan/PEO (1:0.33) fibrils with smooth surfaces. Scale bars: 500 nm.

Chitosan/PEO blended at a ratio of 1:0.33 was electrospun into randomly oriented fibrils that are similar to the collagen fibril meshwork in native AC. Figure 2(a) shows the collagen fibril meshwork in human AC, whereas Figure 2(b) shows electrospun chitosan/PEO fibrils, which were defect-free with smooth surface morphologies. The fibrils exhibited a uniform geometry and clear boundaries between the fibrils. The average fibril diameter estimated from approximately 50 fibrils was 99 ± 23 nm, twofold larger than the collagen fibrils in cartilage.

**Figure 3.**
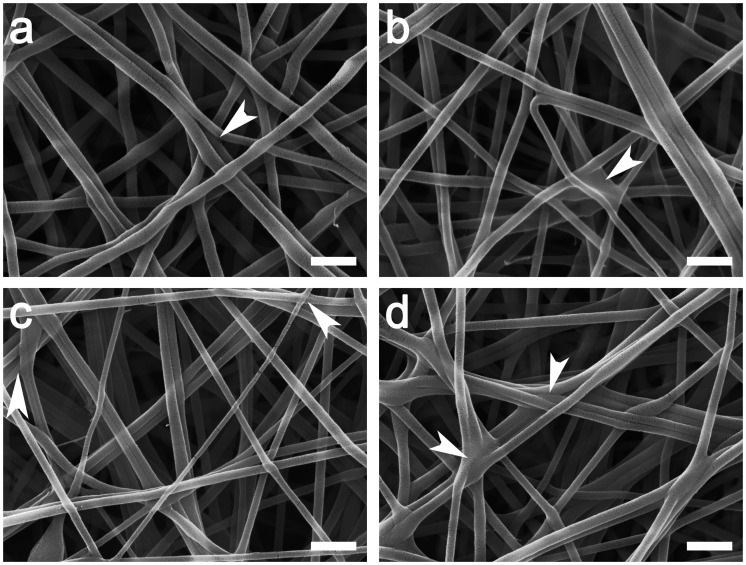
SEM micrographs comparing electrospun chitosan/PEO fibrils crosslinked with different concentrations of genipin: (a) 0.5%, (b) 0.8%, (c) 1.0%, and (d) 1.5% genipin. Adjacent fibrils were fused together, forming bundle of fibrils. Crosslinks between some fibrils can be observed as indicated by the white arrow heads. Scale bars: 500 nm.

**Figure 4.**
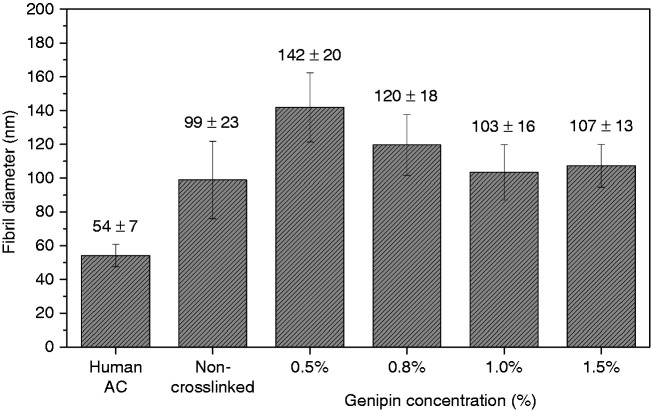
Average diameters of electrospun chitosan/PEO fibrils without crosslinking, and after crosslinking with various concentrations of genipin, in comparison with the diameter of collagen fibrils in human AC.

The as-spun chitosan-PEO fibrils were stable in dry state, but they rapidly dissolved when transferred to an aqueous solution. Therefore, crosslinking was required to improve the water resistance of chitosan/PEO scaffolds. After electrospinning, the chitosan/PEO scaffolds were crosslinked with different concentrations of genipin solutions, ranging from 0.5%, 0.8%, 1.0% to 1.5%. This yielded scaffolds which remained undissolved upon contact with aqueous solution, indicating that crosslinking conferred water resistance to the chitosan/PEO fibrils. Figure 3 shows the structure of the crosslinked fibrils, with some of the adjacent fibrils fused together. All the crosslinked fibrils were swollen after crosslinking, indicated by an increased fibril size, and there was a higher degree of swelling when lower concentration of genipin was used. Before crosslinking, the average diameter of the as-spun chitosan/PEO fibrils was 99 ± 23 nm. The average fibril diameter increased by 43% after crosslinking with 0.5% genipin, 21% with 0.8% genipin, 4% with 1.0% genipin and 8% with 1.5% genipin, shown in Figure 4.

### Stiffness of chitosan/PEO nanofibrous scaffolds

**Figure 5.**
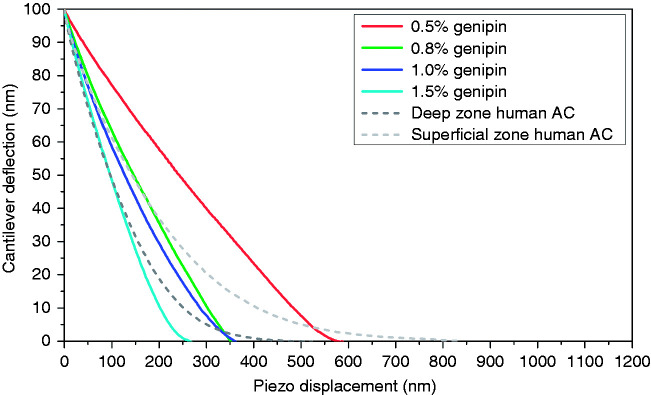
The unloading part of the load-displacement curves measured on chitosan/PEO scaffolds crosslinked with different concentrations of genipin crosslinker, in comparison with that measured on the superficial zone and deep zone human AC.

**Figure 6.**
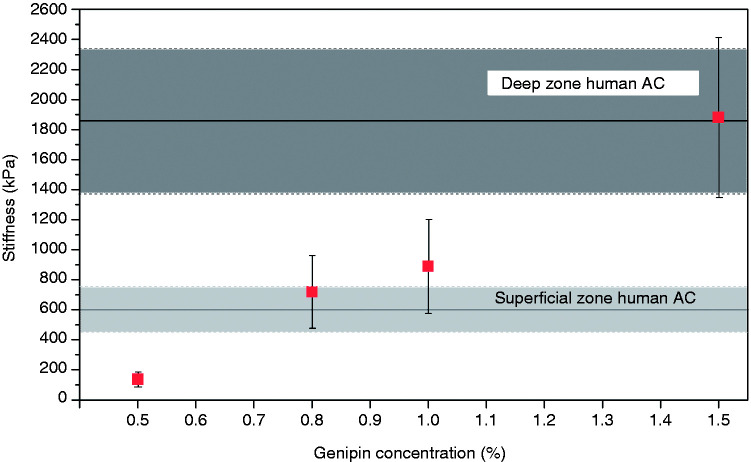
Stiffness of the chitosan/PEO scaffolds in comparison with the stiffness of superficial zone and deep zone human AC. The stiffness of scaffolds increases with the increasing concentration of the genipin crosslinker. The area in light grey shows the stiffness measured on the superficial zone human AC (600 ± 150 kPa), whereas the area in dark grey shows the stiffness measured on the deep zone human AC (1854 ± 483 kPa).

Using spherical indenters with a radius of 5 μm, the overall stiffness of the various structural elements composing AC and the stiffness properties of electrospun fibril meshwork were measured with IT-AFM. The stiffness of superficial zone AC was measured on the original cartilage surface, whereas deep zone AC was measured after removing the superficial and middle zones (∼2 mm) of the sample. Load-displacement curves were recorded using IT-AFM and the unloading part of the curves are depicted in Figure 5. Using data from the upper 25% of the unloading curves, the stiffness was calculated with Equation (1). As shown in Figure 6, the stiffness of chitosan/PEO scaffolds increased with the increasing concentration of the genipin crosslinker. Scaffold crosslinked with 1.5% genipin exhibited the highest stiffness, 1875 ± 532 kPa, which is similar to the stiffness of deep zone human AC of 1854 ± 483 kPa. The stiffness of 1.0% genipin crosslinked scaffold was 890 ± 311 kPa, in between the stiffness of the deep zone and superficial zone human AC of 600 ± 150 kPa. The 0.8% crosslinked scaffold exhibited a stiffness of 719 ± 240 kPa, which is similar to the superficial zone human AC, while the stiffness of 0.5% crosslinked scaffold was the lowest, recorded at 137 ± 47 kPa.

### Degradation of chitosan/PEO nanofibrous scaffolds

**Figure 7.**
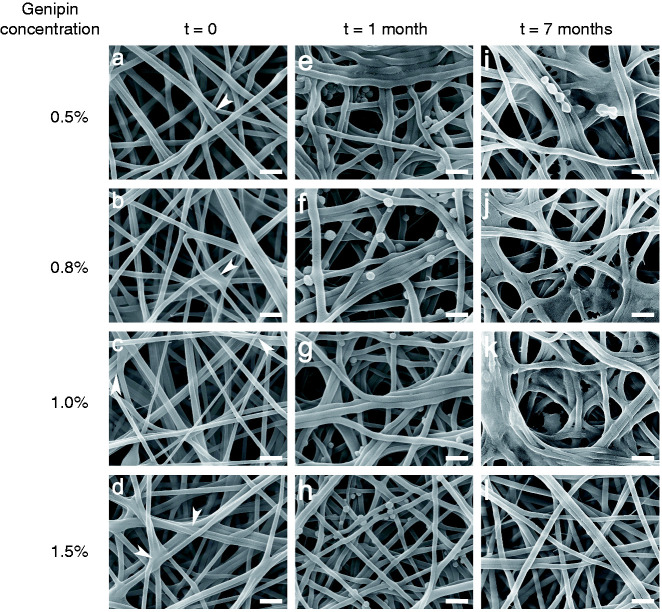
Comparison of SEM micrographs of chitosan/PEO scaffolds before and after hydrolytic degradation. (a-d) The original fibril structure. (e-g) After one month of hydrolytic degradation, some adjacent fibrils were fused together, but (h) the structure of scaffold crosslinked with 1.5% genipin remained unchanged. (i-k) After seven months, the fibrils further dissolved with a loss of fibril structure, but (l) the structure of scaffold crosslinked with 1.5% genipin still remained unchanged. Scale bars: 500 nm.

The column of Figure 7(a) to (d) shows the uniform fibril morphology before degradation and changes of the morphology after one month (Figure 7(e) to (h)) and seven months of degradation (Figure 7(i) to (l)). The rows show the electrospun scaffolds crosslinked with increasing concentration of genipin. Signs of degradation were observed on the scaffolds crosslinked with 0.5%, 0.8% and 1.0% genipin. At one month, some adjacent fibrils were fused together. At seven months, some of the fused fibrils had started to dissolve and lost their fibrillar structure. A reduction in mesh size was also visible, which may reduce the overall scaffold volume. As shown in Figure 7(h) and (l), the structure of the 1.5% genipin-crosslinked scaffold remained stable without the signs of degradation.

**Figure 8.**
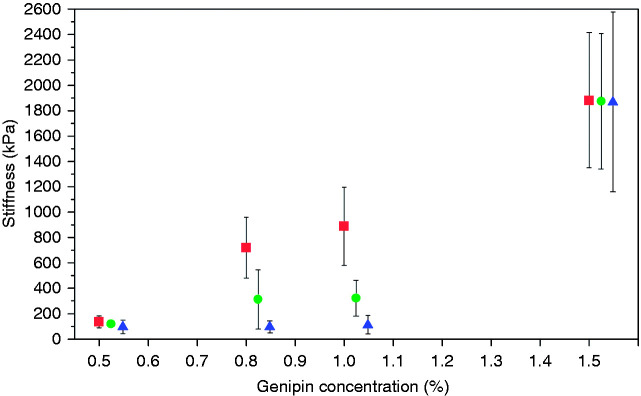
Decrease in the stiffness of chitosan/PEO scaffolds after hydrolytic degradation. The original stiffness of the scaffolds crosslinked with four different genipin concentrations is indicated by “■”. The stiffness of scaffolds after one month of degradation is indicated by “●”, whereas the stiffness after seven months of degradation is indicated by “▲”.

The stiffness of chitosan/PEO scaffolds crosslinked with 0.5%, 0.8% and 1.0% genipin were reduced because of scaffold degradation, as shown in Figure 8. A large reduction of stiffness was observed as early as one month, with 15% reduction on scaffold crosslinked with 0.5% genipin, 57% reduction on that with 0.8% genipin and 64% reduction on that with 1.0% genipin. At seven months, the stiffness of scaffolds crosslinked with 0.5%, 0.8% and 1.0% further reduced to approximately 100 kPa, although their initial stiffness varied. In comparison, the scaffold crosslinked with 1.5% genipin maintained its stiffness throughout the course of the degradation period, with a remaining stiffness of 1865 ± 708 kPa after seven months. This finding is in agreement with the non-changing scaffold structure shown in Figure 7(h) and (l).

### Biocompatibility of chitosan/PEO nanofibrous scaffolds

**Figure 9.**
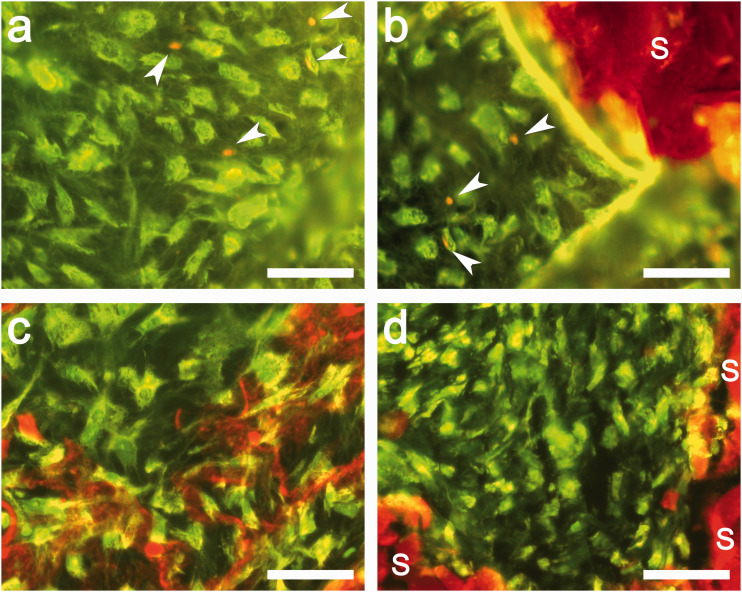
Viability test of HACs seeded on the chitosan/PEO scaffolds. The detected metabolically active cells (green fluorescence) and necrotic cells (red fluorescence, arrow heads) on chitosan/PEO scaffolds crosslinked with (a) 0.5%, (b) 0.8%, (c) 1.0% and (d) 1.5% genipin. The scaffold structure (denoted with “*S*”) was auto fluorescent. The images were taken in a single exposure through FITC long-pass filter appropriate for fluorescein. Scale bars: 50 μm.

Figure 9 shows the fluorescence images of live and dead HACs on genipin crosslinked chitosan/PEO scaffolds. The scaffold fibrils are auto fluorescent, shown by the large area of red fluorochromes that did not exhibit the typical shapes of HACs. This finding has also been confirmed by simultaneously DAPI staining cell nuclei and Cell Tracker Green stained cell cytoplasm. We counted the number of live (green) and dead cells (red) on the confocal images at three different locations on a sample, and obtained a cell viability on the scaffolds of over 90%, with approximately 10% of necrotic cells shown by the red fluorochromes. Only cells located outside the auto-fluorescent area were counted to avoid ambiguous red-fluorescent signals. All chitosan/PEO scaffolds demonstrated a similar high degree of cell viability, regardless of the concentration of genipin crosslinker.

**Figure 10.**
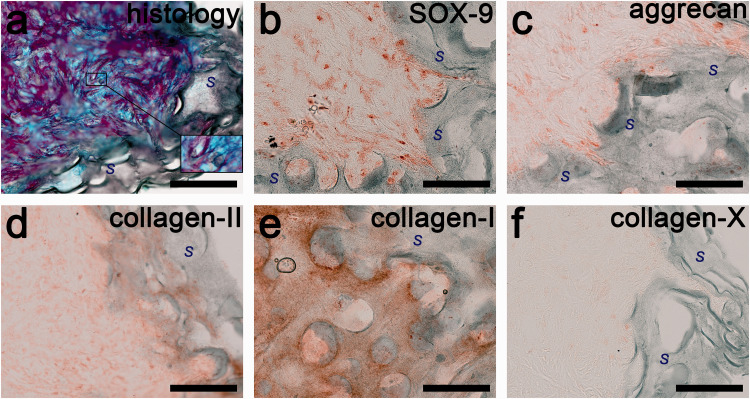
Extracellular matrix formation on HACs-seeded chitosan/PEO scaffold crosslinked with 1.0% genipin. (a) Histology of proteoglycan matrix is shown in blue and the total collagen matrix in red. Cell nuclei are shown in black, with an enlarged image shown in inset. Areas marked with “*S*” are scaffold structure, of which cell nuclei are not observed. Immunohistochemistry of (b) SOX-9, (c) aggrecan, (d) type II collagen, (e) type I collagen, (f) type X collagen with positive reaction detected with the chromogenic AEC substrate and shown in reddish brown colour. Extracellular matrix was absent within the scaffold structure (dark green). Scale bars: 50 µm.

**Figure 11.**
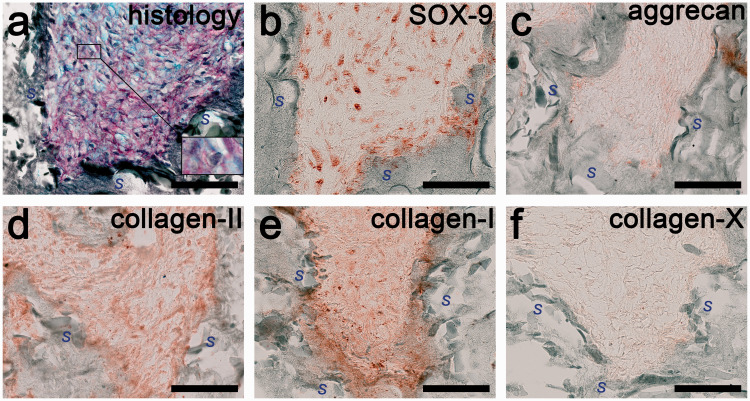
Extracellular matrix formation on HACs-seeded chitosan/PEO scaffold crosslinked with 1.5% genipin. (a) Histology of proteoglycan matrix is shown in blue and the total collagen matrix in red. Cell nuclei are shown in black, with an enlarged image shown in inset. Areas marked with “*S*” are scaffold structure, of which cell nuclei are not observed. Immunohistochemistry of (b) SOX-9, (c) aggrecan, (d) type II collagen, (e) type I collagen, (f) type X collagen, with positive reaction detected with the chromogenic AEC substrate and shown in reddish brown colour. Extracellular matrix was absent within the scaffold structure (dark green). Scale bars: 50 µm.

The biosynthesis and accumulation of extracellular matrix on chitosan/PEO scaffolds crosslinked with 1.0% and 1.5% genipin are shown in Figures 10 and 11, respectively. The genipin crosslinked chitosan/PEO scaffolds are shown by the dark green colour, which surrounds the reddish brown HACs. The inner structure of the scaffolds, however, was not filled with cells (haematoxylin stained) or newly formed extracellular matrix after three weeks. The cells were agglomerated on the surface of the scaffolds and did not penetrate the voids between fibrils. Moreover, histological staining of proteoglycan (Alcian blue stained) and total collagen (Sirius red stained) contents were mainly observed among the cell agglomerates outside the scaffold structure (Figures 10(a) and 11(a)). By visible inspection in the light microscope, the intensity of the histological staining of both proteoglycan and total collagen content in the scaffold crosslinked with 1.0% genipin (Figure 10(a)) was higher than that in the scaffold crosslinked with 1.5% genipin (Figure 11(a)), which indicates a higher amount of extracellular matrix in the 1.0% genipin crosslinked scaffold.

A considerable amount of the chondrogenic differentiation marker, SOX-9, was also detected in the cell agglomerates on both constructs (Figures 10(b) and 11(b)). The expression level of the cartilage specific proteoglycan, the aggrecan, was higher on the 1.0% genipin crosslinked scaffold shown in Figure 10(c) compared to the 1.5% genipin crosslinked scaffold shown in Figure 11(c). Our finding agrees with our histological analysis, which showed a more intense proteoglycan content on the 1.0% genipin crosslinked scaffold. As shown in Figures 10(d) and 11(d), the expression level of type II collagen on both constructs was lower compared to the expression level of type I collagen shown in Figures 10(e) and 11(e). This indicates a fibrous characteristic of the repair tissue. Based on Figures 10(f) and 11(f), the presence of type X collagen is negligible on both constructs.

## Discussion

### Structure of chitosan/PEO nanofibrous scaffolds

The addition of non-ionogenic and flexible super-long-chain PEO macromolecules plays a crucial part in enhancing the chain entanglements to form stable solid fibrils. Molecular interactions between chitosan and PEO are formed by hydrogen bonds between the amino groups in chitosan and the ether groups in PEO. These bonds disrupt the self-association among chitosan chains caused by the strong hydrogen bonding between their NH_2_ and OH groups, which generally result in a highly viscous chitosan solution. When increasing the amount of PEO, the overall viscosity of the polymer solution is decreased monotonically, which allows the formation of a miscible polymer blend system,^36^ and hence, stretches the polymer fibrils efficiently during the electrospinning process to produce continuous and bead-free fibrils. We also added co-solvents, such as DMSO and Triton X-100, into the solution to improve the electrospinning conditions and increase the fibril yield. DMSO relaxes chain entanglement of chitosan, this results in higher structural uniformity in fibrils.^35^ Trace amounts of Triton X-100 (0.3%) act as a non-ionic surfactant so that an improved fibrous structure could be obtained at a high chitosan-to-PEO ratio, which is desirable for cartilage tissue engineering applications.

To increase the stability of chitosan/PEO scaffolds in physiological conditions, genipin was used as the crosslinking agent because it exhibits lower cytotoxicity compared to other synthetic crosslinking agents and may also provide high crosslinking efficiency. During the crosslinking reaction, a covalent bond is formed between the nitrogen from the amino group of chitosan and the carbon from the carbonyl group of genipin. As a result, there is an interconnected network that can restrain chain slippage and increase the stiffness and stability of the scaffolds.^42^ After crosslinking, some of the fibrils were fused to form bundles of fibres, as shown in Figure 3. All scaffolds showed an increased fibril diameter after crosslinking. When a lower genipin concentration was used, a higher degree of swelling was observed (Figure 3). This is attributed to the higher proportion of water in the genipin solution of lower concentration.^31^ As a result of swelling, the pore size of the scaffolds reduced, which, in turn reduced cell penetration into the scaffold.

### Stiffness of chitosan/PEO nanofibrous scaffolds

The genipin crosslinker is effective in regulating the mechanical properties of scaffolds. By using a range of genipin concentrations to crosslink chitosan/PEO, scaffolds of the same chemistry but with a range of crosslinking degrees were produced. A higher concentration of genipin solution resulted into higher crosslinking between fibrils and an increase of scaffold stiffness as shown in Figure 6. However, the increase of stiffness only works up to a threshold concentration, beyond which there will be an increase in the fibril size and crosslinking reaction that is limited at the scaffolds surface, and results in a reduction of stiffness.^29^ Furthermore, it is important to obtain a balance between maintaining high water content for cell viability and exhibiting adequate mechanical integrity to restore the function. Therefore, we chose the highest genipin concentration of 1.5% for crosslinking the chitosan/PEO in this study. In general, the stiffness of chitosan/PEO nanofibrous scaffolds in this study (∼890 kPa for scaffold crosslinked with 1.0% genipin) is lower compared to the chitosan/PEO cast films (∼2000 kPa for film crosslinked with 1.0% genipin) reported in the literature.^11,43^ Such a variation is not unexpected, since there are distinct differences in the structure in both cases. Nonetheless, scaffolds crosslinked both with 1.0% and 1.5% genipin exhibited stiffness values comparable to the native cartilage.

### Degradation of chitosan/PEO nanofibrous scaffolds

Although crosslinking with genipin yields interatomic and intermolecular bonding, the physical and chemical structure of the crosslinked scaffolds is still susceptible to hydrolytic degradation.^44^ Indeed, chitosan with deacetylation >85% has been reported to exhibit a degradation rate of several months *in vivo*, which is considered suitable for cartilage repair purposes.^12,45^ The degradation rate of genipin crosslinked scaffolds is inversely related to the genipin concentration ^34,46^ as the introduction of more crosslinker molecules into the polymer backbone hinders the attack of water molecules. Under the effect of α-MEM medium at 37°C, chitosan/PEO crosslinked with 0.5%, 0.8% and 1.0% genipin exhibited a loss of structural integrity, e.g., fibrils appeared swollen and dissolved, thus forming a reduced mesh size of the scaffolds (Figure 7) as the crosslinking of chitosan/PEO might be incomplete for lower genipin concentrations. In comparison, the scaffold crosslinked with 1.5% genipin retained its structural integrity. This complies well with the stiffness measurements of the scaffolds, in which the stiffness of the scaffolds with degraded structures reduced, while the scaffold with an unchanged structure did not show lower stiffness. Scaffolds with a higher degree of crosslinking were more stable.

### Biocompatibility of chitosan/PEO nanofibrous scaffolds

A porous structure with interconnected pores is essential in the design of scaffolds towards AC repair to provide the necessary space for cell adhesion and matrix growth. Electrospinning of chitosan/PEO produced nanofibrous scaffolds that exhibited a porous network, but the high density of the nanometre-sized electrospun fibrils resulted in a denser packing of fibrils and smaller mesh size compared to the meshwork found in AC. The smaller mesh size prevents the cells from penetrating the voids between the fibrils, and depositing new extracellular matrix within the scaffolds. Consequently, extracellular matrix formation was mainly observed at the surface of the scaffolds. In addition, an increasing crosslinking density had caused the fibrils to become brittle, which further limited subsurface migration of cells, and hence, cell proliferation and proteoglycan synthesis.^34,46,47^ Figures 10(a) and 11(a) show that the extracellular matrix formation on the 1.0% genipin crosslinked scaffold was higher than that on the 1.5% genipin crosslinked scaffold, which may be a direct consequence of the differences in the scaffolds’ mesh sizes. A smaller mesh size of the higher crosslinked scaffold (1.5%) also results in a slower diffusion of physiological nutrients and may reduce cell proliferation and physically lower cell expansion. Previous research also reported that chondrocytes within PEG hydrogels of higher crosslinking density exhibited decreased cell proliferation, which was associated with a lower total DNA content.^47^ In the higher crosslinked and stiffer gels, the chondrocytes were less metabolically active and sterically impeded from the increase in cell diameter.^48^ In addition, the non-degradable behaviour of the 1.5% genipin crosslinked scaffold also obstructs the ingrowth of the extracellular matrix.

Our analysis of extracellular matrix formation, which unfortunately was fibrous-like, exhibiting a higher content of type I collagen rather than the formation of type II collagen. The newly formed tissue on the scaffold crosslinked with 1.5% geninpin looked more fibrous-like compared to that crosslinked with 1.0% genipin, suggesting that a higher crosslinking density exerts an effect on the de-differentiation of chondrocytes.

## Conclusion

Electrospinning of chitosan/PEO followed by crosslinking with genipin produced nanofibrous scaffolds that closely mimic the structure and stiffness of the collagen fibril meshwork in native AC. When increasing the concentration of genipin, we observed smaller fibril diameters and an increase in the stiffness of the scaffolds. Crosslinking the chitosan/PEO scaffold with 0.8% and 1.0% genipin resulted in a stiffness of the polymer meshwork similar to the superficial zone of human AC of 600 ± 150 kPa, whereas the stiffness of scaffold crosslinked with 1.5% genipin was comparable to the stiffness of deep zone cartilage of 1854 ± 483 kPa. Following our biocompatibility assays, we did not observe significant signs of degradation, even after seven months, in the scaffold crosslinked with 1.5% genipin. However, we observed degradation of structure and stiffness when the concentration of genipin crosslinker was lower than 1.0%. Due to the high fibril density of the nanometre-sized fibrils, the scaffolds exhibited mesh sizes that were relatively smaller than the size of HACs so the extracellular matrix formation was mainly concentrated on the scaffold surface. We conclude that chitosan/PEO scaffolds crosslinked with approximately 1.0% genipin were appropriate for cell proliferation and tissue regeneration. As an improvement of the electrospinning method, we proposed wet electrospinning, which allows electrospun fibrils to deposit in an aqueous solution, to solve the problem with the overly dense fine fibrils as it will allow more equal cell seeding and proliferation. Taken together, this work is a step further towards tailoring the structure and stiffness of scaffolds to produce engineered cartilage for healing cartilage defects in patients and to avoid secondary osteoarthritis and joint replacement surgery.
